# Dr. Merle Cornelius Newkirk

**Published:** 1916-01

**Authors:** 


**Dr. Merle Cornelius Newkirk,** Johns Hopkins 1913, of Sherman, died at Ellicottville, November 24, aged 28.

